# Timing of 17-hydroxyprogesterone measurement during the standard-dose Synacthen test in pediatric patients evaluated for non-classical congenital adrenal hyperplasia due to 21-hydroxylase deficiency

**DOI:** 10.20945/2359-4292-2026-0081

**Published:** 2026-07-27

**Authors:** Nurdan Çiftci, Emine Çamtosun, İsmail Dündar, Edip Ünal, Nurullah Çelik, Ayşehan Akıncı

**Affiliations:** 1 Konya City Hospital, Department of Pediatric Endocrinology, Konya, Turkey; 2 İnonu University Faculty of Medicine, Department of Pediatric Endocrinology, Malatya, Turkey; 3 Dicle University Faculty of Medicine, Department of Pediatric Endocrinology, Diyarbakır, Turkey; 4 Sivas Cumhuriyet University Faculty of Medicine, Department of Pediatric Endocrinology, Sivas, Turkey; 5 Başkent University Faculty of Medicine, Department of Pediatric Endocrinology, Ankara, Turkey

**Keywords:** 17-hydroxyprogesterone, Synacthen, congenital adrenal hyperplasia, 21-hydroxylase deficiency

## Abstract

**Objective:**

We aimed to evaluate the diagnostic utility of the 30-minute 17-hydroxyprogesterone (17-OHP) measurement during the standard-dose Synacthen test.

**Subjects and methods:**

This retrospective study analyzed the medical records of patients aged 0-18 years who underwent Synacthen testing for suspected non-classical congenital adrenal hyperplasia due to 21-hydroxylase deficiency between 2000 and 2024.

**Results:**

Among 150 patients included, the median age was 13 years (range 5.5-18), and 85.3% were female. Twenty-nine patients exhibited a peak stimulated 17-OHP level ≥ 10 ng/mL. In three of these patients, the 30-minute 17-OHP level was within normal limits, whereas all had diagnostic 17-OHP levels at 60 minutes. No patients exhibited a diagnostic 17-OHP level at 30 minutes with a non-diagnostic value at 60 minutes. Using a basal 17-OHP cut-off value of 3.78 ng/mL, sensitivity and specificity were 89.7% and 94.2%, respectively. The V281L variant was the most frequently identified pathogenic variant.

**Conclusion:**

The 60-minute 17-OHP measurement demonstrated greater diagnostic sensitivity than the 30-minute measurement in this cohort and may provide adequate diagnostic information in pediatric patients evaluated for non-classical congenital adrenal hyperplasia, as no additional cases were identified exclusively at the 30-minute time point.

## INTRODUCTION

The short (standard-dose) Synacthen test, also known as the corticotropin stimulation test, is the gold standard for diagnosing primary adrenal insufficiency. Serum cortisol and 17-hydroxyprogesterone (17-OHP) levels are analyzed at baseline and at 30 and/or 60 minutes following corticotropin administration (125 µg for patients < 2 years of age and 250 µg for those >2 years of age), preferably as an intravenous bolus, with a peak cortisol level <18 µg/dL indicating adrenal insufficiency (^1^,^2^). A baseline or stimulated 17-OHP concentration >100 ng/mL is diagnostic for classical congenital adrenal hyperplasia due to 21-hydroxylase deficiency (21-OHD-CAH). In the non-classical form (NC-CAH), basal 17-OHP is generally between 2 and 100 ng/mL (^2^). However, the assessment of basal, 30-minute, and/or 60-minute cortisol and 17-OHP concentrations using the standard-dose Synacthen test when 17-OHP is < 100 ng/mL is an established practice used to confirm the diagnosis of 21-OHD-CAH (^2^). Several publications recommend measuring cortisol and 17-OHP at 0 and 60 minutes in the Synacthen test, while in the United Kingdom, sampling at 30 minutes is also recommended (^3^).

Comparison of basal and stimulated 17-OHP levels usually allows differentiation between normal individuals, heterozygotes, patients with NC-CAH, and patients with classical CAH, though overlapping values exist across these groups (^4^). A basal 17-OHP < 2 ng/mL and a stimulated 17-OHP < 10 ng/mL exclude CAH with high probability, while a stimulated 17-OHP of 10-100 ng/mL supports the diagnosis of 21-OHD NC-CAH (^2^,^4^).

Several studies have evaluated the adequacy of the 60-minute cortisol value alone in demonstrating cortisol insufficiency during short or standard-dose Synacthen tests, finding the 60-minute value sufficient (^5^-^7^). To our knowledge, no studies in children compare 17-OHP levels at 30 and 60 minutes in the standard-dose Synacthen test for diagnosing 21-OHD CAH. Therefore, this study sought to assess the diagnostic contribution of the 30-minute 17-OHP measurement compared with the 60-minute value in pediatric patients undergoing the standard-dose Synacthen test for suspected 21-OHD non-classical congenital adrenal hyperplasia (NC-CAH). Omitting the 30-minute measurement, while not reducing total test duration, could decrease the number of blood samples, thereby improving patient comfort and reducing costs.

## SUBJECTS AND METHODS

In this retrospective study, we analyzed the medical records of patients aged 0-18 years who were admitted to the Pediatric Endocrine Departments at İnönü University, Cumhuriyet University, and Dicle University Medical Faculties between 2000 and 2024. Included patients presented with hirsutism, early axillary and/or pubic hair growth, menstrual irregularities, acne, a basal laboratory 17-OHP level ≥ 2 ng/mL, and/or advanced bone age, and underwent Synacthen testing with a pre-diagnosis of 21-OHD NC-CAH. Classical forms (salt-wasting or simple virilizing) of 21-OHD were excluded. Salt wasting CAH due to 21-hydroxylase deficiency typically presents early in life and is fatal if untreated. The simple virilizing form may present with pronounced bone age advancement, clitoromegaly in girls, and macrogenitalia in boys. Patients with clinical and genetic features of classical 21-OHD-CAH were excluded.

Patients with other enzymatic defects, such as 11β-hydroxylase deficiency, 3β-hydroxysteroid dehydrogenase deficiency, or POR deficiency, may have elevated 17-OHP levels. Individuals with symptoms indicative of these defects (e.g., hypertension, ambiguous genitalia, skeletal deformities, elevated 11-deoxycortisol) and/or genetic results inconsistent with 21-OHD NC-CAH were excluded. Blood sampling for adrenal hormone analysis was performed at 08:00. In menstruating girls, samples were taken during the early follicular phase (between days 3 and 5 after the onset of spontaneous bleeding). None of the patients had taken medication potentially interfering with hormone testing, such as oral contraceptives, gonadotropin-releasing hormone analogues, metformin, or cortisone, for at least three months prior to sampling.

Basal cortisol and 17-OHP levels were measured at baseline. A 250 µg intravenous bolus of Synacthen was then administered, and stimulated cortisol and 17-OHP concentrations were measured at 30 and 60 minutes post-administration. 17-OHP and cortisol levels were analyzed using the MAGLUMI 2000 (Shenzhen New Industries Biomedical Engineering Co., China) fully automated chemiluminescent immunoassay analyzer. A peak stimulated cortisol <18 µg/dL was considered inadequate (^1^); a peak 17-OHP ≥ 10 ng/mL was suggestive of 21-OHD NC-CAH (^4^). For patients with a peak 17-OHP ≥ 10 ng/mL, the timing of this value was recorded, as were the genetic analysis results.

Patients were stratified into two groups based on peak 17-OHP during Synacthen testing: Group 1 (biochemically suggestive of NC-CAH; peak 17-OHP ≥ 10 ng/mL) and Group 2 (controls; peak 17-OHP < 10 ng/mL). Baseline hormone test results were compared between groups. To address potential biochemical overlap between NC-CAH and heterozygous carriers, *CYP21A2* genetic analyses within Group 1 were retrospectively reviewed. The genetic testing methods and corresponding molecular results were systematically recorded. The study protocol was approved by the local ethics committee.

### Statistical analysis

Statistical analyses were performed using SPSS for Windows (v. 25.0, SPSS, USA). The normality of quantitative data was assessed with the Kolmogorov-Smirnov test and histogram plots. Two-group comparisons were performed using the Mann-Whitney U test. Receiver operator characteristics (ROC) curves were generated to determine sensitivity and specificity of baseline 17-OHP. Numerical data are presented as median and range, qualitative data as number (percentage), with *p* < 0.05 considered statistically significant.

## RESULTS

Of the 150 patients analyzed, the median age was 13 years (5.5-18), and 128 (85.3%) were female. The most common presentation was early axillary and/or pubic hair growth (n = 70, 46.7%), followed by hirsutism (n = 52, 34.7%), menstrual irregularity (n = 20, 13.3%), acne (n = 5, 3.3%), and precocious puberty (n = 3, 2%). Median baseline DHEA-S was 207.5 µg/dL (14.9-864); median baseline 17-OHP was 1.68 ng/mL (0.15-57.4); median 30-minute 17-OHP was 2.815 ng/mL (0.15-70.1); median 60-minute 17-OHP was 3.16 ng/mL (0.15-80).

Twenty-nine patients (Group 1) exhibited peak 17-OHP ≥ 10 ng/mL during Synacthen testing. In 26 of these, both 30- and 60-minute 17-OHP levels were ≥ 10 ng/mL. Three patients had normal 30-minute 17-OHP, while 60-minute 17-OHP was ≥ 10 ng/mL in all three. No patient had a 60-minute 17-OHP <10 ng/mL with a 30-minute value ≥ 10 ng/mL. In Group 1, median 30-minute 17-OHP was 17.20 (1.9-70.10) ng/mL; median 60-minute 17-OHP was 25 (10.1-80.0) ng/mL. There was no statistically significant difference between median 30- and 60-minute levels.

The median age of Group 1 was 9.8 (5.5-17.6) years, and Group 2 was 13 (5.6-18) years; this difference was not statistically significant (*p* = 0.389). Fifteen (51.7%) of 29 patients in Group 1 and 57 (47.1%) of 121 in Group 2 were prepubertal. Median basal DHEA-S in Group 1 was 184 (14.9-572) µg/dL, and in Group 2 209 (15-864) µg/dL (*p* = 0.148). Median basal 17-OHP in Group 1 was 9.32 (0.74-57.4) ng/mL; in Group 2 it was 1.42 (0.15-7.56) ng/mL (*p* < 0.001).

**Figure 1:**
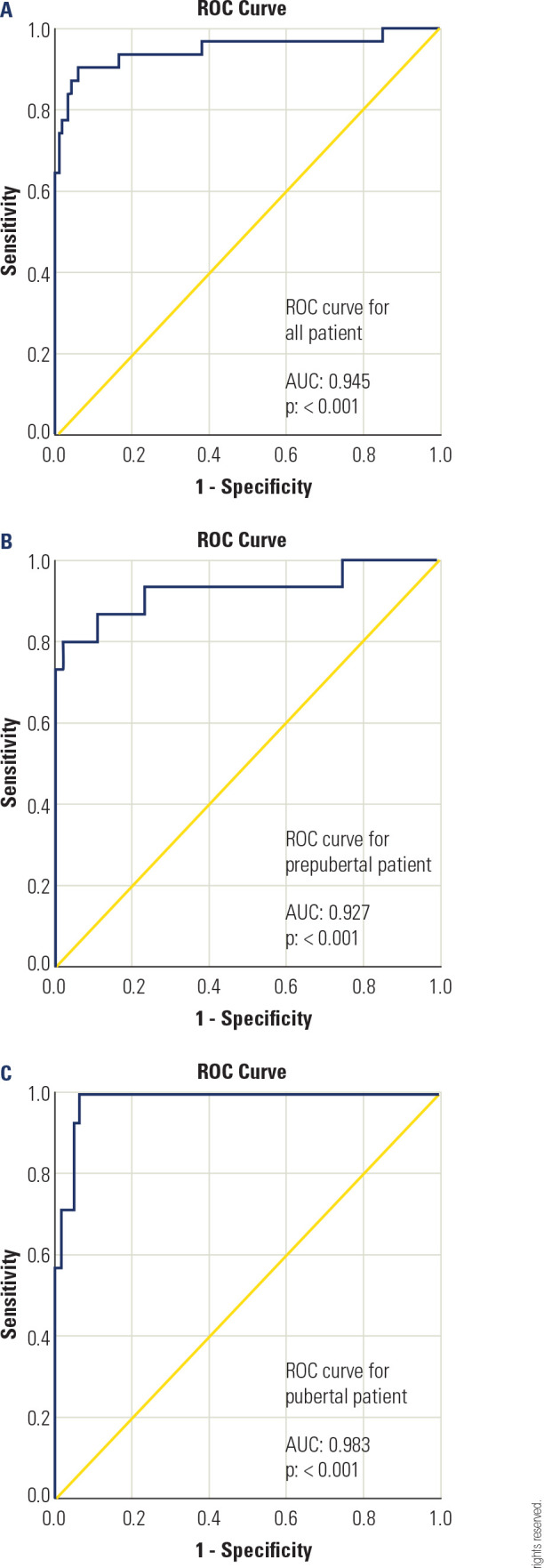
ROC analysis of basal 17-OHP level in predicting standard dose Synacthen test positivity and summary of analysis. (**A**) All patients group; (**B**) Prepubertal patients group; (**C**) Pubertal patients group.

ROC analysis demonstrated that basal 17-OHP significantly predicted a positive test result (*p* < 0.001, **Figure 1**). Using 3.78 ng/mL as the cut-off for basal 17-OHP, sensitivity was 89.7% and specificity was 94.2%. The patients were stratified by pubertal status (pre-pubertal versus pubertal). The optimal cut-off values identified by ROC analysis for the groups were 2.525 and 3.85 ng/mL for the prepubertal and pubertal groups, respectively. These resulted in sensitivity and specificity values of 86.7% and 89.5% (prepubertal) and 92.86% and 93.75% (pubertal), respectively. Using the commonly recommended cut-off of 2 ng/mL (^4^), sensitivity was 93.1% and specificity 68.6% for the whole group; sensitivity was 86.7% and specificity 80.7% for prepubertal, and sensitivity 100% with specificity 57.8% for pubertal children (**Figure 1**).

A peak cortisol ≥18 µg/dL was considered an adequate response. In Group 1, 11 of 29 patients had an inadequate cortisol response. Of the 18 with adequate cortisol, 15 had both 30- and 60-minute responses ≥18 µg/dL; three had 30-minute cortisol <18 µg/dL but 60-minute ≥18 µg/dL. No patients had 60-minute cortisol <18 µg/dL but a 30-minute value ≥18 µg/dL. In Group 2, no patients exhibited inadequate peak cortisol (**Table 1**). *CYP21A2* molecular analysis was performed in 21 of 29 Group 1 patients: 9 (42.8%) had biallelic mutations (homozygous or compound heterozygous), 6 (28.5%) were isolated heterozygous carriers, and 6 (28.5%) had no mutations detected using available methodologies (**Table 1**).

**Table 1:** Patients with peak 17-OHP ≥ 10 ng/mL

Patient	Sex	Age at presentation	Complaint	Basal ACTH	Basal DHEA-S		17-OHP				Cortisol			Genetic analysis	Genotype variation	Phenotype
							Basal	30-min	60-min		Basal	30-min	60-min			
		(years)		pg/mL	mcg/dL		-----ng/mL-----				-----mcg/dL-----					
P1	F	7.5	Pubic hair	37.1	86.7		22.3	24.4	24.8		4.4	9.64	12.3	Whole gene sequence analysis	IVS2-13C>G heterozygousExon 7 V281L heterozygous	NC-CAH
P2^*^	F	6	Pubic hair	17.6	88.7		11	25	25		10.9	20	22	NGS-PCR and MLPA	In2G(C>G) homozygous	NC-CAH
P3^*^	M	9.8	Pubic hair	22.8	310		9	14.9	19.2		8.57	13.7	16.3	NGS-PCR and MLPA	In2G(C>G) homozygous	NC-CAH
P4	F	9.25	Pubic hair	131	23.3		23.9	30.2	32		10	15.2	17.5	Whole gene Exon sequence analysis and intron 2	Exon 7 V281L homozygous	NC-CAH
P5	F	15.75	Hirsutism	30.7	25.8		50	70.1	80		9.4	10.6	11.8	Whole gene Exon sequence analysis and intron 2	Exon 7 V281L heterozygousExon 4 I172N heterozygous	NC-CAH
P6	F	5.5	Pubic hair	30	15		2.67	10.2	10.1		11.38	20.16	22.4	Whole gene Exon sequence analysis and intron 2	Exon 7 V281L heterozygous	NC-CAH
P7^*^	F	7.88	Pubic hair	74.7	91		57.4	58.8	67.7		9.2	9.5	10.4	Whole gene Exon sequence analysis and intron 2	Exon 7 V281L heterozygous	NC-CAH
P8^*^	F	7.88	Pubic hair	105	68.1		12.5	15.9	16.2		9.9	10.9	11.2	Whole gene Exon sequence analysis and intron 2	Exon 7 V281L heterozygous	NC-CAH
P9	F	15.3	Hirsutism	57.6			15.2	28.4	41.2		15.4	16.8	20.1	Whole gene sequence analysis	Exon 7 V281L homozygous	NC-CAH
P10	M	13.2	Acne	57	444		6.87	22.4	26.5		11.1	14.4	15.6	Whole gene sequence analysis	Exon 7 V281L homozygous	NC-CAH
P11	F	6.5	Pubic hair	67	57		20	63.8	70.8		16.5	18.3	19.9	Whole gene sequence analysis	Exon 4 I172N heterozygousExon 7 V281L heterozygous	NC-CAH
P12	F	7.5	Pubic hair	32	90		13.6	23.1	71.6		12.1	16.3	17.4	Whole gene Exon sequence analysis and intron 2	Exon 10 P453S homozygous	NC-CAH
P13	F	14	Hirsutism	29.3	282		9.1	11.7	13.4		18	22.8	27.4	Whole gene Exon sequence analysis and intron 2	Exon 10 A434V homozygous	NC-CAH
P14	F	13.5	Hirsutism	41.5	572		5	25	32		8.3	10.8	11.4	NGS-PCR	Exon 8 Q318X heterozygous	NC-CAH
P15	F	8.83	Pubic hair	72.8	77.2		5.59	35	36.9		10.6	26	31	NGS-PCR	Exon 7 V281L homozygous	NC-CAH
P16	F	16.3	Hirsutism	56.3	450		27	24	50		11.4	11	9.85	Whole gene sequence analysis	None detected	NC-CAH
P17	F	13.7	Hirsutism	19.5	279		3.93	13.1	17.3		14	17.8	21	Whole gene sequence analysis	None detected	NC-CAH
P18	F	8.11	Precocious puberty	13.2	15		6.96	29.14	35.48			17.2	19.8	-	None detected	NC-CAH
P19	F	9.8	Pubic hair	85	166		0.74	1.9	31.4		18.74	26.5	29.4	NGS-PCR	None detected	NC-CAH
P20	F	14.11	Hirsutism	20.9	523		9.18	13.38	18.72		10.3	24.7	32.2	-	None detected	NC-CAH
P21	M	7	Pubic hair	25	212		1.77	3.39	13		13	25.9	34	Whole gene sequence analysis	None detected	NC-CAH
P22	F	17.6	Hirsutism	20	408		5	17.2	17.7		11.7	20.2	22.5	Not tested	-	NC-CAH
P23	F	16.5	Hirsutism	60.1	184		11.82	12.87	12.34		19.8	22.9	24.6	Not tested	-	NC-CAH
P24	F	7	Pubic hair	182	34.5		9.32	10.38	11.7		23.8	30.8	31.8	Not tested	-	NC-CAH
P25	F	16.1	Hirsutism	16	191		9.47	13.32	13.72		14.9	23.8	27.4	Not tested	-	NC-CAH
P26	F	17.2	Hirsutism	18.5	558		3.8	4.41	65.37		28	21.2	24.5	Not tested	-	NC-CAH
P27	F	17.11	Hirsutism	25	391		12.43	16.38	16.9		13.5	19.6	23.6	Not tested	-	NC-CAH
P28	F	14.4	Menstrual irregularity	13.3	161		6.01	10.71	12.07		12.6	18	24.6	Not tested	-	NC-CAH
P29	M	9.1	Pubic hair	99.2	-		50.9	60.1	63.9		12	13.1	13.2	Not tested	-	NC-CAH

ACTH: adrenocorticotropin hormone; F: female; M: male; adrenocorticotropin hormone; DHEA-S: dehydroepiandrosterone sulfate; 17-OHP: 17-hydroxyprogesterone; NGS-PCR: next-generation sequencing-polymerase chain reaction; MLPA: multiplex-ligation-dependent-probe-amplification. NC-CAH: non-classical congenital adrenal hyperplasia.

* P2 and P3 are siblings; P7 and P8 are twins.

## DISCUSSION

Non-classical congenital adrenal hyperplasia due to 21-hydroxylase deficiency is approximately ten times more prevalent than classical CAH, with clinical features well recognized in women but often missed in men, contributing to the gender imbalance observed in many studies (^8^-^11^). In the present study, 81.8% of genetically confirmed 21-OHD NC-CAH (homozygous and compound heterozygous) and 86.2% of those with Synacthen test peak 17-OHP ≥10 ng/mL were female.

Comparing the 29 patients with peak 17-OHP ≥ 10 ng/mL and the 121 with < 10 ng/mL, no differences in age or basal DHEA-S were observed, whereas basal 17-OHP was significantly higher in those testing positive. Similar findings were reported by Gönç and cols. (^12^), where basal 17-OHP was higher among patients with peak Synacthen 17-OHP ≥ 10 ng/mL, while DHEA-S was not significantly different. Using 3.78 ng/mL as the cut-off for basal 17-OHP, sensitivity and specificity were 89.7% and 94.2%, respectively. In similar studies conducted in Turkey, Sahmay and cols. (^13^) determined the cut-off value of basal 17-OHP to be 2.25 ng/mL (50.4% sensitivity and 84% specificity) and Cengiz and cols. (^14^) reported it to be 3.19 ng/mL (75% sensitivity and 51.6% specificity). The specificity of the current study’s cut-off was superior for predicting a positive test. Using the commonly accepted 2 ng/mL cut-off, sensitivity was found to be 93.1% with a specificity of 68.6% in predicting a positive test result. In our study, the optimal cut-off value was 2.525 ng/mL in prepubertal patients and 3.85 ng/mL in pubertal patients, and specificity increased with the use of these values without a significant decrease in sensitivity. Nonetheless, screening tests are generally biased towards sensitivity, as it is deemed more important not to miss a diagnosis, at the cost of accepting lower specificity.

Raising the cut-off value increases the specificity of this test at the expense of lower sensitivity, although some patients with the condition can be missed. Therefore, based on our findings, we cannot confirm whether the cut-off value should be increased. Additionally, given the lack of a uniform genetic gold standard across all patients and the potential overlap between NC-CAH and heterozygous carriers, these ROC-derived cut-off values should be interpreted as exploratory and hypothesis-generating rather than definitive for clinical practice.

Previous studies assessing cortisol response at 30 and 60 minutes during the standard-dose Synacthen test have shown that 60-minute cortisol levels were adequate (≥ 18 µg/dL) in patients with inadequate at 30 minutes, with no case being found in which the 30-minute cortisol response was adequate while the 60-minute cortisol response was not (^5^-^7^). In these studies, 4.8-8.6% of patients could be diagnosed with cortisol insufficiency and incorrectly treated if a decision was made based on the 30-minute value alone, and it therefore possible to conclude that the 60-minute cortisol value should definitely be measured and may be sufficient for diagnosis (^5^-^7^).

In the present study, among patients with peak 17-OHP ≥ 10 ng/mL, 18 had an adequate cortisol response; in three, only the 60-minute value was sufficient, while in 15 both measurements were sufficient. Notably, all patients with elevated peak 17-OHP had 60-minute 17-OHP ≥ 10 ng/mL, with three having normal 30-minute values.

Thus, our results support the conclusion that the 60-minute cortisol and 17-OHP measurements more reliably capture the peak hormonal response than the 30-minute measurement. While omitting the 30-minute measurement does not reduce the overall test duration, it significantly improves clinical efficiency by reducing the number of invasive blood samples required. In the pediatric population, minimizing invasive procedures is essential for improving patient comfort and reducing procedure-related anxiety. Eliminating unnecessary intermediate measurements also reduces healthcare costs and laboratory workloads.

Nevertheless, our observation that the 60-minute measurement detected all positive cases is based on a relatively small cohort of 29 patients with elevated 17-OHP. This sample size may have limited the statistical power to definitively conclude that an early peak at 30 minutes does not occur in NC-CAH. Hence, despite the 60-minute measurement being more reliable, the results may not be generalizable to all clinical settings without further validation in larger populations.

In terms of genetic findings, the V281L pathogenic variant was the most common genotype among our 21-OHD NC-CAH patients. This findings is persistent with the literature, as V281L variations have been reported as the most common genetic variation (34-55.9%) in the *CYP21A2* gene in patients diagnosed with 21-OHD NC-CAH in different studies conducted in Turkish, Spanish, Greek, and French populations (^15^-^18^). The V281L variant was homozygous in four patients, compound heterozygous in three, and heterozygous in another three. As further genetic analysis (e.g., MLPA) was not performed in the patients with heterozygous variation, compound heterozygosity could not be excluded, suggesting patients may be NC-CAH or symptomatic carriers. Evidence has also indicated peak 17-OHP levels in 21-OHD CAH carriers in the ranges of 4-10 or 5-15 ng/mL (^19^,^20^).

We acknowledge that using a peak 17-OHP threshold of 10 ng/mL may include some heterozygous carriers, as seen in six of our tested patients. The overlap between symptomatic heterozygotes and NC-CAH patients is a recognized diagnostic challenge, particularly with immunoassays (e.g., chemiluminescent immunoassay), which may overestimate 17-OHP compared to LC-MS/MS. However, the primary focus of this study was to assess the 30- versus 60-minute temporal dynamics of the peak response rather than to establish new diagnostic criteria.

This study has several limitations. Its retrospective design and the relatively small number of positive Synacthen test results may have limited generalizability. Patient selection based on clinical judgment may have also introduced bias. Genetic confirmation was not available for all patients, and genetic analyses utilized different methods (e.g., PCR, NGS, and MLPA) over an extended period. These methods vary in sensitivity, particularly for large deletions or complex rearrangements in the *CYP21A2* gene. Lastly, while MLPA was used in some cases to increase the detection rate of deletions, the lack of a standardized genetic protocol for all patients may have led to an underestimation of some variants.

In conclusion, our findings suggest that the 60-minute 17-OHP measurement may capture the peak response in most pediatric patients evaluated for suspected 21-OHD NCCAH. However, prospective studies with larger cohorts and comprehensive genetic confirmation are required before definitive modifications of current testing protocols can be recommended. If further validated, this approach may reduce testing costs, decrease laboratory workload, and improve patient comfort.

## Data Availability

the datasets used and/or analyzed during the current study are available from the corresponding author upon reasonable request.
